# No date for the PROM: the association between patient-reported health events and clinical coding in primary care

**DOI:** 10.1186/s41687-020-0183-5

**Published:** 2020-03-02

**Authors:** Paul J. Barr, Scott A. Berry, Wendolyn S. Gozansky, Deanna B. McQuillan, Colleen Ross, Don Carmichael, Andrea M. Austin, Travis D. Satterlund, Karen E. Schifferdecker, Lora Council, Michelle D. Dannenberg, Ariel T. Wampler, Eugene C. Nelson, Jonathan Skinner

**Affiliations:** 1 The Dartmouth Institute for Health Policy & Clinical Practice, Dartmouth College, One Medical Center Drive, Lebanon, NH 03756 USA; 2 Institute for Health Research, Kaiser Permanente Colorado, 10065 E. Harvard Ave, Denver, CO 80231 USA; 3 Dartmouth-Hitchcock Nashua, 2300 Southwood Drive, Nashua, NH 03063 USA; 4 Geisel School of Medicine at Dartmouth College, Hanover, NH 03755 USA

**Keywords:** Patient outcomes, Falls and injuries, Elderly, primary care/general practice

## Abstract

**Objective:**

It is unclear whether data from patient-reported outcome measures (PROMs) are captured and used by clinicians despite policy initiatives. We examined the extent to which fall risk and urinary incontinence (UI) reported on PROMS and provided to clinicians prior to a patient visit are subsequently captured in the electronic medical record (EMR). Additionally, we aimed to determine whether the use of PROMs and EMR documentation is higher for visits where PROM data was provided to clinicians.

**Design:**

We conducted a cross-sectional patient-reported risk assessment survey and semi-structured interviews with clinicians to identify themes related to the use of PROMs.

**Setting:**

Fourteen primary care clinics in the US (eight intervention and six control clinics), between October 2013 and May 2015.

**Participants:**

Primary care clinicians and older adult (≥66 years) patients completing a 46-item health risk assessment, including PROMs for fall risk and UI.

**Intervention:**

Risk assessment results provided to the clinician or nurse practitioners prior to the clinic visit in intervention clinics; data was not provided in control clinics.

**Main outcome:**

1) Agreement between ICD-9 codes of fall risk or UI in the EMR and patient-reports, and 2) clinician experience of PROMs use and impact on coding.

**Results:**

A total of 505 older adult patients were included in the study, 176 at control clinics and 329 at intervention clinics. While patient reports of fall risk and UI were readily captured by PROMs, this information was only coded in the EMR between 3% – 14% of the time (poor Kappa agreement). Intervention clinics performed slightly better than control clinics. Clinician interviews (*n* = 16) revealed low use of PROMs data with multiple barriers cited including poor access to data, high quantity of data, interruption to workflow, and a lack of training on PROMs.

**Conclusions:**

Current strategies of providing PROMs data prior to clinic visits may not be an effective way of communicating important health information to busy clinicians; ultimately resulting in underuse. Better systems of presenting PROMs data, and clinician training on the importance of PROMs and their use, is needed.

## Introduction

Over 50% of the symptoms and signs of illness go undetected during clinic visits [1, 2]**.** Patients often report symptoms earlier and more frequently than documented by clinicians; thus clinicians may be missing the opportunity to intervene early before the patient’s condition deteriorates [3]**.** This failure to address reported symptoms is especially problematic for older adults with multi-morbidity, where early identification of deteriorating conditions is essential to avoid exacerbation of the medical problem and resultant hospitalizations [4]**.** Patient-reported outcome measures (PROMs) have been identified as a way of “harnessing patients’ voices to improve clinical care” by systematically capturing important information on physical and mental health [3]**.** Data generated from PROMs can potentially change the process of care, including increased education, counseling, referrals, and number of diagnoses made during the visit [5]**.** While evidence of improved health-related outcomes is mixed, [5, 6] recent results in cancer [7] and primary care settings [8] shows that the use of PROMs use in routine care improved health outcomes.

The use of PROMs, once restricted to the research world, is now promoted by current health policy initiatives [9]**.** Most recently, the Medicare Merit-Based Incentive Payment System (MIPS) and Advanced Alternative Payment Models (APMs) both included the use of PROMs as metrics underpinning quality of care payment models in the U.S. [10] Data from PROMs are now routinely collected at many institutions during annual wellness visits (AWV), with the aim of informing clinical care by identifying symptoms early, monitoring health, and enabling the creation of personalized prevention plans for patients [9, 11]**.**

Yet it remains unclear whether PROMs data is routinely used by clinicians to guide clinical care [9, 12]. The implementation of PROMs adds additional complexity to already burdened clinicians, while also introducing additional work for the patient. Logistical concerns exist over how to administer the PROMs, and the sheer number of potential PROMs to assess [9]**.** Measurement challenges also exist regarding the determination of clinically significant scores. A greater concern is that the collection of PROMs data is an exercise in regulatory compliance, reducing the likelihood of data usage during an already busy clinic schedule [9]**.** One potential solution to assisting physicians in using PROMs results is to include them in the electronic medical record (EMR), and other salient formats. Theoretically, if these results are readily available, physicians would be more likely to review and use these results during clinical visits.

The aim of this project is to assess whether providing PROMs results on fall risk and urinary incontinence (UI) to primary care clinicians resulted in the increased captured of this data in the EMR (ICD Codes), which we hypothesize to reflect higher use of this data during the clinic visit. We focused on patient reports of fall risk and UI as these issues are highly prevalent, are accurately identified by patients, and are amenable to improvement once identified [13, 14]**.**

## Methods

### Design & Setting

Data for this manuscript comes from a multi-site prospective cohort study, with control clinics. Patients were recruited from a convenience sample of fourteen primary care clinics between October 2013 and May 2015. Clinics choosen included clinical champions, advocates for the project, in order to increase the likelihood of clinician support and project success: four from Dartmouth-Hitchcock (D-H) in NH (two intervention and two control clinics) and ten from Kaiser Permanente (KP) in CO (six intervention and four control clinics). PROMs data were collected from patients and provided to clinicians prior to the visit. At the D-H site intervention clinics, the PROMs data were provided within the EMR report. At the KP intervention clinics, a personal prevention plan (see Supplementary Material 1) report reflecting PROMs data was printed by clinic staff and provided on paper to clinicians. At both D-H and KP intervention sites, a note would appear in the clinician’s scheduling program indicating the need to review PROMs data. Clinicians were asked to review the PROMs data and either act on it during the visit or encourage the patient to follow up with appropriate care staff. In control clinics at both KP and D-H, while PROMs data was completed by patients, this data was not provided to clinicians.

Clinicians received basic information about the goals of the project and use of PROMs data. To determine the extent of PROMs use we: 1) compared the presence of ICD-9 codes as a proxy for the discussion of PROMs data and 2) conducted a series of semi-structured interviews with clinicians to understand how and if they used PROMs data. The Dartmouth Committee for the Protection of Human Subjects (#00024060) and the Kaiser Permanente Colorado Institutional Review Board (CO-12-1734) approved this research.

### Participants

Participants were eligible if they were ≥ 66 years of age, attending a clinic visit, spoke English, and were continuously enrolled in a health plan at each clinic for the preceding 12-months. Patients were excluded if they were unable to provide informed consent, had a history of substance abuse, were unable to complete PROMs due to physical or cognitive impairment, were enrolled in hospice care or were a permanent resident at a skilled nursing facility, or had completed an AWV within the prior ten months. For the semi-structured interviews, eligible clinicians were those with patients that had completed both baseline and 12-month follow-up health risk assessments (HRAs).

### Data

Patients completed an HRA via a patient portal, telephone, or in clinic on an electronic tablet within seven days prior to their initial visit. The HRA consisted of 46 PROMs, including domains of functional status (e.g. fall risk, UI – see Table 1), health behaviors, mental health, and socio-demographics. The electronic medical record (EMR) was reviewed by nursing staff for all International Classification of Disease (ICD-9) codes related to fall risk or UI, within seven days prior to and 60 days post the patient’s clinic visit. This timeframe allows for clinicians who do not complete coding immediately after the clinic visit to complete their coding prior to the study’s data collection. We used ICD codes as an indication of discussion the problem during the clinic visit, as such information is typically captured in a problem list, which must conform to standardized vocabularies based on ICD and SNOMED codes as part of the Centers for Medicare and Medicaid Services (CMS) Meaningful Use initiative [15].

**Table 1:** Falls and UI health risk assessment items

Item	ICD-9 codes
Urinary Incontinence	
Many people experience problems with urinary incontinence, the leakage of urine. In the last 6 months have you accidentally leaked urine? (Yes|No)	788.2788,20,788.21,788.3788.31, 788.32,788.3788.30,788.33,788.34, 788.35,788.36,788.37,788.38,788.39, 788.63,788.69,788.91,788.43,600.01
Falls^a^	
K-P - A fall is when your body goes to the ground without being pushed. Did you fall in the past 12 months? (Yes|No) DH & DCS - In the past 12 months have you fallen? (Yes|No)	V15.88, 781.2, 781.3,and all E880.x through E888.x
K-P - In the past 12 months, have you had a problem with balance or walking? (Yes|No) DH & DCS - In the past 12 months, have you experienced difficulties with balance or walking? (Yes|No)	

^a^A patient was considered to be at risk of falls if they selected a ‘yes’ response to either fall question

At both KP and D-H, it was not mandatory for patients in the control clinics to have a scheduled visit following the completion of their HRA. To reduce the risk of selection bias, we only included individuals who visited the clinic within 31 days of HRA completion, excluding those who did not visit the clinic during this time period.

### Clinician interviews

An experienced qualitative researcher (TDS) completed semi-structured interviews with clinicians from both intervention and control clinics, to gather a deeper understanding of how PROMs data were used. Staff from each clinic identified eligible clinicians in a two-month period when interviews were being conducted. A list of patients who had completed both baseline and 12-month follow-up HRAs and were scheduled for a visit in the two-month window was generated by clinic staff. Research staff then randomly sampled one patient per provider and asked clinic staff to schedule a block of time (30–40 min) for a telephone interview within three days of the patient’s visit. This allowed us to explore both visit-specific events, including potential use of the HRA results, along with general use and views of PROMs (see Supplementary Material 2).

### Analysis

Kappa coefficients were calculated to determine the chance-corrected agreement between patient reports of fall risk or UI and clinician diagnostic codes in the EMR [16]. We hypothesized moderate to high agreement in intervention clinics, reflecting higer use of the PROMs data, and low agreement in control clinics. Baseline differences between patients in the intervention and control clinics were compared using Students T-Tests and Chi-Square Tests. Analyses were conducted in Stata, version 14 (StatCorp); *p*-values < 0.05 were considered statistically significant. Interviews were recorded, transcribed, and analyzed using content analysis [17] to determine emergent themes by two coders (TS, KS).

## Results

Of 432 potential participants at D-H clinics and 686 at KP clinics, 352 patients at D-H (81%; 188 control, 164 intervention) and 302 patients at KP (44%; 111 control, 191 intervention) were eligible and agreed to take part. Of those enrolled, 123 patients from control sites (116 patients at D-H and 7 patients at KP) and 26 patients from intervention sites (5 patients from D-H and 21 from KP) had no visit within 31 days of HRA survey completion and were excluded. A total of 505 patients were included in the analysis (Table 2). Most participants in the control and intervention clinics were white, with high levels of education and income. Patients from intervention clinics were slightly older (75 years on average) than those from control clinics (73 years on average) and had a higher Charlson Co-Morbidity Index (3.3 compared to 2.7).

**Table 2:** Patient demographics (*n* = 505)

	Control (*n* = 176)	Intervention (*n* = 329)
Age^a^, Mean (SD), years	72.9 (5.5)	75.1 (6.4)
Gender (%)		
Male	81 (46%)	159 (48%)
Female	95 (54%)	170 (52%)
Race/Ethnicity (%)		
● White	157 (89%)	299 (91%)
● Non-White	19 (11%)	30 (9%)
Education^b^ (%)		
● Less than college	49 (28%)	111 (34%)
● Graduated college	45 (26%)	55 (17%)
● Post graduate	55 (31%)	84 (25%)
● Missing	27 (15%)	79 (24%)
Income^a^ (%)		
● $15,000 - $35,000	15 (8%)	20 (6%)
● $35,000 - $50,000	24 (14%)	74 (22%)
● $50,000 - $75,000	40 (23%)	167 (51%)
● >$75,000	97 (55%)	68 (21%)
Charlson Co-Morbidity Index^a^ (SD)	2.7 (1.6)	3.3 (1.9)
PROMIS: Global Mental Health Score (SD)	53.2 (8.5)	52.1 (9.9)
PROMIS: Global Physical Health Score (SD)	47.6 (9.2)	48.8 (8.2)

^a^*p* < 0.0001; ^b^*p* < 0.05

### Agreement between patient reports and diagnostic codes at the visit

Patient-reported fall risk ranged from 36% to 47% and symptoms of UI from 43% to 44% across clinics. However, clinicians’ coding of fall risk and UI in the EMR was low in control clinics, ranging from 3% to 4%, and 4% to 9%, respectively, revealing a poor-to-slight kappa agreement with patient-reports (Table 3). Clinicians at intervention clinics coded only slightly more patient reports of fall risk (8% vs. 5%) and UI (14% vs. 3%), than those at control clinics (Table 3).

**Table 3:** Kappa Agreement between PROMs and electronic medical record

	Incidence of patient-report *N* (%)	Incidence of diagnostic code *N* (%)	Kappa agreement statistic (95% CI)	Sensitivity ^b^	Specificity ^c^
Intervention clinics (*n* = 329) ^a^					
Falls *N* = 318	115 (36%)	13 (4%)	0.04 (−0.01, 0.10)	0.08	0.98
UI *N* = 325	142 (44%)	29 (9%)	0.10 (0.03, 0.17)	0.14	0.95
Control clinics (*n* = 176) ^a^					
Falls *N* = 174	81 (47%)	5 (3%)	0.07 (0.01, 0.14)	0.05	0.99
UI *N* = 173	75 (43%)	7 (4%)	−0.03 (−0.09, 0.04)	0.03	0.88

^a^ Missing fall data from 11 individuals from intervention clinics and 2 from control clinics, missing UI data from 4 individuals from intervention clinics and 3 from control clinics

^b^ Sensitivity of a clinical test refers to the ability of the test to correctly identify those patients with the disease. Calculated by *true positives/(true positive + false negative)*

^c^ Specificity of a clinical test refers to the ability of the test to correctly identify those patients without the disease. *Calculated by true negatives/(true negative + false positives)*

### Interviews with clinicians

A total of 16 clinicians, 10 from D-H and 6 from KP (12 females, 4 males) were interviewed across sites with an average of 20.5 years (range = 9–31 years) practicing medicine. Not all KP clinician interviews could be scheduled within three days of a study participant’s visit.

### Use of PROMs in practice

Clinicians held positive views about the concept of PROMs, because they were viewed as allowing better preparation for the visit, and a more prioritized discussion of medical issues. They also felt that the collection of PROMs data was “streamlined” due to the use of newer technology (e.g., “tablets”.) However, the majority highlighted significant barriers that limited their use of PROMs data during the clinic visit. At both KP and D-H intervention clinics, clinicians initially described using PROM data to guide their patient interactions. However, upon further probing, clinicians revealed that they would typically make a “quick scan” of PROMs reports (e.g., “it’s rare that I spend more than about 10 seconds”). Clinicians also reported that the sheer quantity of PROMs data made it difficult to use (e.g., “it’s more information than I would want, too much” and “a lot of that information is unnecessary”). When clinicians did use the PROMs data, they would typically do so to “make sure there weren’t any red flags”. As one clinician described:

*“I will literally skim it for 10 seconds while I’m in the room with them. And I’m looking more just for red flags that I have to address. Unfortunately, with the wellness visits there’s a lot of stuff we have to cover and during that time I don’t have any time to [use it] in a meaningful way, and review the physical activity and all the diet questions that get asked. And I don’t have time to use that. So, it’s unfortunate that that information can’t be used better but there are so many requirements that we have, for Meaningful Use, and the annual wellness visits to cover other preventive things that it’s just too much information in the time we’re allotted.”*

Clinicians at KP felt that the printed “patient prevention plan” form provided to the physician was “easy to use”, and reported that if there was little time to discuss data with patients, they would direct patients to look at the print-out and follow through with direction on it. However, the KP clinicians also reported a “drop off” in the number of printed forms provided for use in the clinic visit compared to when the project started. Clinicians at D-H received “pop-up alerts” in the EMR generated by responses to PROMs. However, the data were not well integrated into the EMR and clinicians had to “click on different tabs and scroll through a bunch of pages to find” what they wanted, resulting in an “incredibly inefficient” search for further information on PROMs data, which was “time-consuming” and “frustrating”. This appeared to be the result of a poor user-interface and lack of training in the use of PROMs. As one clinician described:

*“So, you know it’s a challenge if I’m in a visit mode and I’ve got other patients to see, if this information is not in a way that’s easy for people to get at, then it’s difficult. And you know it’s just a lot of clicks and certainly a lot of it is me not probably being as good as I could be with the [EMR] to find this stuff easily, but it shouldn’t be this complicated.”*

Perhaps the best indicator of the PROMs’ utility—or lack thereof—can be derived from the physicians’ patient visits with the study participants at the D-H site, as most interviews were conducted immediately after a patient visit with a study participant. In each of these cases the physicians confided that very little patient information was gleaned from the contents of the PROMs prior to, or during the patient visit; several reported not reviewing the PROMs data at all. One clinicians mentioned difficulty in accessing the data via the EMR, one felt it was not necessary to review the PROMs as it consists of “the same information that I ask about”, and another stated s/he never did unless s/he received an email from the patient portal ahead of the visit.

### Coding PROMs data

When asked specifically about ICD-Coding, clinicians often did not input a diagnostic code based on PROMs, unless it was something of significant concern for the clinician or something that might lead to significant changes in ongoing treatment. For example, most clinicians contended that if something like a UI or a recent fall (or series of falls) came up in their review of PROMs, they may discuss it with the patient, but rarely if ever would they code it. As one clinician stated:

“ *… if I was able to have a discussion separately about that issue with the patient, then I would code it as such … I’d put it in their problem list. But if we didn’t have time and the patient never mentioned anything to me about it, … I’d give … him or her the AVS [after visit summary] and the letter [personal prevention plan]. I wouldn’t always code that, which maybe is not the right thing to do, but I didn’t code it because we didn’t really talk about it.”*

Additionally, diagnostic coding or transference of PROMs to a patient’s EMR was often predicated on follow-up care in which the clinicians could provide further medical attention in the form of medication or a referral:

*“I think if the patient says it’s a minor problem … and I’m not going do anything about it [give a referral], I wouldn’t necessarily code it.”*

Other significant reasons for lack of coding included: 1) respondents never receiving explicit instructions to code based on PROMs data; as such, they did not consider coding as part of their primary responsibilities; and 2) systems not being set up to easily input diagnostic codes based on the PROMs results (full qualitative report is available upon request).

## Discussion

Despite the promise of PROMs, patient-reported data provided in advance of the clinic visit for fall risk and UI were not coded by clinicians across 14 primary care clinics in two large health systems. Similar to prior research, semi-structured interviews revealed multiple barriers to the use and documentation of PROMs data, including challenges in accessing the data, the high quantity of data, interruption to workflow, and lack of clinician training on the use of PROMs [5, 6].

Our findings support previous research that finds a lack of agreement between illness’ signs and symptoms reported by patients and coresponding documentation by clinicians: [1–3] clinicians tend to under-recognize and under-report symptoms experienced by patients [18]. While PROMs are considered a strategy to formalize the reporting of clinically important and actionable symptoms, clinicians in our study missed opportunities to intervene. Even in comparison to clinics where clinicians did not receive PROMs data, the use of PROMs did little to improve the coding of the data into the patient’s electronic records.

While this project demonstrated that patients are willing to engage with PROMs ahead of their clinic visits, clinicians reported a lack of understanding of how to use PROMs data and challenges with accessing the PROMs information in the EMR. Lavallee et al. [9] previously highlighted similar logistical and technological barriers to the use of PROMs data by clinicians. EMRs are not primarily designed for the storage of patient-reported data in discrete data fields, limiting the ability of clinicians to access, interpret, and act upon the data. While the use of PROMs printed and provided to clinicians was considered an improvement by clinicians, the quantity of data still resulted in a quick “scan”. Additionally, clinicians in this study did not appear to place significant value on PROMs data, which has also been observed in previous case studies as a significant and modifiable barrier to PROM use in practice [12].

The process of integrating PROMs into the health system and more rigorous training may be key to overcoming their lack of use [19–21]. It has been theorized that PROMs data can improve patient outcomes by revealing information on patient symptoms to clinicians that may otherwise not be discussed, promoting diagnostic accuracy, clinical intervention and documentation [21]. Yet, inadequate training of clinicians on PROMs, their validity and their use can act as a barrier to successful implementation of PROMs [21, 22].

Beyond clinician training, an alternative strategy to the delivery of PROMs data used in the current study, where data is only provided to clinicians for review, is to engage other health care professionals in the monitoring of patients and use of this data. This strategy was used by Basch et al. [7] in a recent trial where patients were randomized to 1) receive a follow-up call from case managers if symptoms reported on PROMs were severe or worsening, or 2) have their PROMs data integrated into the clinic visit for clinician review only—similar to the strategy in the current project. Overall survival rates were five-months longer for patients randomized to the case manager group, with early responsiveness to worsening symptoms identified as a possible mechanism explaining these differences. This finding suggests the need to move beyond the traditional sporatic use of PROMs during clinic visits to incorporate them into routine practice while involving additional members of the care team.

The current research is not without limitations. The project was implemented at the clinic level, with consent of clinic leadership, not individual clinicians. As such, we did not collect data on the number of clinicians who had a patient take part in the project, or their characteristics. It is possible that clinicians discussed the PROMs data during the visit and decided it did not necessitate coding. However, epidemiological data support the incidence of both falls [23] and UI [24] reported by patients, suggesting underreporting by clinicians in this project. Alternatively, clinicians may have entered information on the discussion in the ‘free-text’ visit summary or ‘problem list’, rather than a diagnostic code; prior research finds that minor symptoms are less likely to be coded, but still may be documented in the EMR [25]. However, based on interviews, it remains more likely that the lack of coding reflects a lack of discussion of the clinical matter. A lack of coding or adequate storage of PROMs data can also reduce continuity of care, as it is more difficult for other clinicians to monitor changes in patient care. For example, it would be important for a clinician to be aware that a patient is at risk of falls if considering prescribing a sedative or antidepressant, as these medications would increase fall risk [26].

## Conclusion

It’s not enough to just measure PROMs; they also need to be integrated into clinical care. Simply providing PROMs data to clinicians at the beginning of office visits is not an effective strategy for communicating this information, as it never appears to make it into the patient’s electronic record, thus limiting the potential of PROMs to impact patient care. There is a need to more effectively use PROMs data in practice; otherwise both patients and health systems will fail to reap the benefits of this potentially valuable investment.

## Supplementary information

**Additional file 1.** Example of a tailored personalized prevention plan at KP.

**Additional file 2.** Clinician interview guide.

## Data Availability

The datasets used and/or analyzed during the current study are available from the corresponding author on reasonable request.

## Abbreviations

APMs: Advanced Alternate Payment
AWV: Annual wellness visit
CO: Colorado
D-H: Dartmouth-Hitchcock
EMR: Electronic medical record
HRA: Health risk assessment
ICD: International Classification of Disease
KP: Kaiser Permanente
MIPS: Medicare Merit-Based Incentive Payment System
NH: New Hampshire
PROMs: Patient-reported outcome measures
UI: Urinary incontinence
