# Supplementary material for: No date for the PROM: the association between patient-reported health events and clinical coding in primary care
Source: J Patient Rep Outcomes. 2020 Mar 2;4:17. doi: 10.1186/s41687-020-0183-5 (PMC7052084; doi:10.1186/s41687-020-0183-5)
Supplement: Supplementary file 1 — Additional file 1. Example of a tailored personalized prevention plan at KP. [file 41687_2020_183_MOESM1_ESM.docx]

**Example of Tailored PPP**

At six months, members will receive a personalized paragraph at the top of their PPP that highlights the changes. The normal PPP will appear below that.

Here is an example of a member who has had a nutrition change for the worse, but a tobacco change for the better.

Dear Mickey,

We reviewed the results of the health survey you completed. We noticed changes since last time in your nutrition and tobacco usage. For nutrition, you may not be getting a balanced diet. Please contact KP Nutrition Services (kphealthyme.com or call 303-614-1070) if you need help maintaining good nutrition. For assistance with other food resources, please call the Hunger Hotline at 855-855-4626 toll free or 720-382-2920. For tobacco, we are so glad to see you've given up using tobacco! Quitting tobacco products is one of the single biggest things you can do for your health. Hopefully you've already started to notice an improvement in how you feel. Keep up the good work! Please let us know if you need any further support or information.

Below is your personalized prevention plan to remind you of available resources. At Kaiser Permanente, we’re not just here to take care of you when you’re sick. We want to keep you healthy and thrive!

If you have any questions or concerns, consider scheduling a phone visit with your provider to discuss further.

Thank you,

Dr. Mouse and the IHR research team

***Personal Prevention Plan****:*

**Physical Activity**: We are happy to know that you are active, which reduces many health risks. For ideas to help you slowly increase your physical activity to 150 minutes of exercise weekly (for example, doing three 10 minute blocks each day on 5 days each week), please go to kp.org/healthyaging or call Silver Sneakers at 1-800-476-2167 (TTY 1-866-513-9964).

**Mental Health:** Please talk to your health care team about any emotional health (such as depression and anxiety) problems. In addition to talking with your health care team, go to kp.org/depression for self care tips or call member services at 303-338-3800 (TTY: 303-338-3820) and ask for Behavioral Health options. For Southern Colorado, call 1-888-681-7878 (TTY: 1-800-521-4874).

**Nutrition:** you may not be getting a balanced diet. Please contact KP Nutrition Services (kphealthyme.com or call 303-614-1070) if you need help maintaining good nutrition. For assistance with other food resources, please call the Hunger Hotline at 855-855-4626 toll free or 720-382-2920.

**Falls**: Please talk to your health care team if you have had a recent fall or balance concerns. Many health problems can increase your risk of falling. You may find helpful ways to reduce your fall risk by visiting kp.org/watch and click on No More Falls to watch a fall prevention video. For information about Fall Prevention classes, call 1-866-868-7112 for times, cost, and locations.

**Template with changes**

Dear [patient name],

We reviewed the results of the health survey you completed. We noticed changes since last time in your [changed variable] and [changed variable]. For [first variable], [insert change language from six month protocol] For [next variable], [insert change language from six month protocol]

Below is your personalized prevention plan to remind you of available resources. At Kaiser Permanente, we’re not just here to take care of you when you’re sick. We want to keep you healthy and thrive!

If you have any questions or concerns, consider scheduling a phone visit with your provider to discuss further.

Thank you,

[provider name] and the IHR research team

***[Paste Personalized Prevention plan]***

**Template without changes**

Dear [patient name],

We reviewed the results of the health survey you completed. There were no major changes in any of the measures compared with your last survey.

Below is your personalized prevention plan to remind you of available resources. At Kaiser Permanente, we’re not just here to take care of you when you’re sick. We want to keep you healthy and thrive!

If you have any questions or concerns, consider scheduling a phone visit with your provider to discuss further.

Thank you,

[provider name] and the IHR research team

***[Paste Personalized Prevention plan]***
