# Supplementary material for: No date for the PROM: the association between patient-reported health events and clinical coding in primary care
Source: J Patient Rep Outcomes. 2020 Mar 2;4:17. doi: 10.1186/s41687-020-0183-5 (PMC7052084; doi:10.1186/s41687-020-0183-5)
Supplement: Supplementary file 2 — Additional file 2. Clinician interview guide. [file 41687_2020_183_MOESM2_ESM.doc]

**PRO Provider Interview Guide**

**Greeting:** I understand that your practice has been involved with implementing the use of patient reported outcome (PRO) measures. These may be the pre-visit surveys that patients complete online or a survey done in the waiting room.

**Purpose**: The purpose of this interview is to learn about how these patient reported outcome (PRO) measures are implemented, to see if and how these measures/reports are used, and if so, to better and also get your overall perceptions of them. Additionally, we want to specifically gain your impressions of your visit with the patient today in using the patient measures derived from the pre-visit survey. **[If no name of specific patient, then speak generically about the last interaction they had when they used the PRO measures—see below.]**

**Confidentiality:** Assure confidentiality and permission to record interview. [No names will ever be included in any reports or anything disseminated.]

****Make sure to record.** ☺

1. First, in terms of better understanding the whole process of using patient reported outcome (PRO) measures, do you mind walking me through the process of how they’re administered and implemented in your office/practice? [i.e. How are you involved with patient reported measures in your practice? Etc.]

**Now I want to talk a little about today’s visit with XXXX [Must have patient info; if not, then try to ask about the last time they used the measures in an interaction with a patient and then ask specifically about that interaction, using questions below.]**

1. How would you say the visit went over all? What were the things that went well and what were the things that you wished had gone better? **[Possible probes: Did PRO measures at all guide your interaction? Anything flagged? Do you think the PRO measures are helpful? Etc.] ***Try to glean specifics about using the PRO measures here.***
2. Was this visit any different than others because of the use of the patient reported outcome (PRO) measures?
3. What was the information you had about the patient and the reason for the visit prior to meeting with the patient? Were you able to use it prior to meeting with him/her?
   1. **Was there information you wished you had that you did not have?**
4. More generally speaking, how difficult or easy is it to access the patient reported outcome measures? [**Possible probes: Integrated into system? Populate into patients’ charts? Ease of software? Specific things that you are looking for? Workflow issues? Time issues? Etc.]**
   1. What was **most useful** about having the information? What was **least useful**? What about more specific data… For example, if a patient filled out on the survey that he or she had urinary incontinence or a recent fall, are you able to find/access this information?  (Probe **if yes:**   What do you do with this information?  Probe about coding (process), depending on answer [If no mention of coding, must ask if they code these types of things; probe].  **If no: Why not?  Probe about process, software and system.**)
   2. Any changes you would recommend for this type of information?
5. **[If not discussed earlier…]**How do you see the **results** from patient reported outcome (PRO) measures? Are you able to use these results/measures? **[Possible probes: Helpful? Beneficial? Efficient? Structure visits? Share with patients? Helpful when addressing sensitive topics? Other ways that the reports are used? Etc.]**
   1. Are there any other ways that the data are used in your practice?
   2. **Challenges with measures/report? [Other challenges or barriers?]**
6. Have you ever been offered the opportunity to provide input about the survey or the implementation of the measures? [**Probe: Have the goals of the patient reported outcome measures been communicated to you? Communication about PROs in general? Communication about best practices for using the data/measures? Etc.]**
7. Do you see the process of using patient reported outcome (PRO) measures as benefitting the patients? How do the patients perceive this process of filling out the surveys and discussing them? **[Probe: Has anything surprised you about the use of patient reported outcome (PRO) measures?]**
8. Given what you have shared, what do you think of patient reported outcome measures and how could they be more helpful for your practice or patient care? [**Any suggestions that you can make to make the process or the report more helpful?]** Improvements made to benefit patients?
